# Quantification of timelapse 3D tumor spheroid killing activity of NK cells using a live-cell imaging system

**DOI:** 10.1371/journal.pone.0334246

**Published:** 2025-10-14

**Authors:** Jakkrapatra Srisantitham, Nontaphat Thongsin, Siriwal Suwanpitak, Methichit Wattanapanitch

**Affiliations:** 1 Siriraj Center for Regenerative Medicine, Research Department, Faculty of Medicine Siriraj Hospital, Mahidol University, Bangkok, Thailand; 2 Department of Immunology, Faculty of Medicine Siriraj Hospital, Mahidol University, Bangkok, Thailand; Sun Yat-Sen University, CHINA

## Abstract

Natural killer (NK) cells are critical components of the immune system, responsible for recognizing and eliminating a wide range of abnormal cells, including those infected by pathogens or transformed into cancerous cells. Their potent cytotoxic functions, encompassing the direct release of cytotoxic granules, antibody-dependent cell cytotoxicity (ADCC), and the expression of apoptosis-inducing ligands, make NK cells a promising therapeutic product in cancer immunotherapy. This study presents a detailed protocol for assessing NK cell-mediated cytotoxicity against three-dimensional (3D) tumor spheroids using a live-cell imaging system, offering a more physiologically relevant model compared to traditional 2D cultures. Utilizing this 3D spheroid model, we explored the dynamics of NK cell killing activity against two aggressive solid cancer cell lines, cholangiocarcinoma (KKU-213A) and triple-negative breast cancer (MDA-MB-231), across varying effector-to-target (E:T) ratios. Our findings reveal a dose-dependent increase in NK cell activity, with higher E:T ratios yielding more pronounced tumor cell death. Real-time imaging further demonstrated distinct differences in the morphology and cell death patterns of the two cell lines, with MDA-MB-231 cells exhibiting a faster response to NK cell cytotoxicity. These findings underscore the utility of 3D spheroid models and live-cell imaging for studying NK cell function and advancing the development of NK cell-based immunotherapies. The standardized protocol detailed herein provides a valuable insights into immune surveillance and therapeutic applications.

## Introduction

NK cells are innate lymphoid cells that play a pivotal role in recognizing and eliminating aberrant cells, including those infected by viruses or bacteria, stressed cells, foreign cells, and tumor cells [1,2]. Their potent cytotoxic capabilities make them attractive for cancer therapy. Mature NK cells can destroy tumor cells by detecting the downregulation of MHC class I or the upregulation of stress ligands. They can kill directly through the release of cytotoxic granules (e.g., perforin and granzyme) [3], initiate ADCC via CD16 recognition, and induce apoptosis by expressing FasL and TRAIL [4,5]. Moreover, NK cells can produce specific cytokines such as IFN-ɣ and GM-CSF, that trigger immune responses of other immune cells [6]. Unlike T cells, NK cells eliminate damaged cells in an HLA-independent manner without prior sensitization. [7]. Additionally, tumor cells normally downregulate MHC class I molecules to escape T cell recognition, allowing these cells to be detected and eliminated by NK cells [8,9]. Previous clinical studies have shown that adoptive NK cell therapy worked effectively in allogeneic settings with low evidence of graft-versus-host disease (GvHD), cytokine release syndrome (CRS), or other toxicities such as immune effector cell-associated neurotoxicity syndrome (ICANS) [10–12]. Consequently, NK cells are considered potential off-the-shelf cell products for cellular immunotherapy.

Cytotoxicity of NK cells can be assessed using several techniques, with co-culturing them with cancer cell lines as one of the most convenient and well-established techniques. However, traditional 2D co-culture systems fall short in replicating the tumor microenvironment (TME) [13]. Tumor spheroid assays, on the other hand, provide a 3D in vitro model that could offer a more physiologically relevant system for studying cell interactions and therapeutic efficacy. These models can incorporate multiple cell types, including tumor cells, stromal cells, and immune cells, enabling the study of complex cell-cell interactions within the TME [14]. The spheroid model also allows the gradients of oxygen, nutrients, and metabolic waste, closely simulating that of heterogeneous conditions within solid tumors, including hypoxic cores and proliferative outer layers [15,16]. By providing a more accurate in vitro model, spheroid assays enable high-throughput screening of treatments, reducing the need for costly animal studies [14].

The morphology of tumor spheroids plays a crucial role in their interaction with immune cells, and real-time imaging can reveal distinct morphological features that influence immune response. For instance, some exhibit a smooth, round shape with well-defined borders, providing a more uniform and organized target for immune cells [16], others may display spike-like protrusions, resulting in a less uniform, irregular surface [17]. These protrusions can hinder immune cells, altering their ability to efficiently target and kill tumor cells [18]. Additionally, tumor budding, a process where isolated or small clusters of cancer cells break away from the primary tumor, can be observed in spheroid models, complicating the immune response [19]. Tumor budding cells are more likely to evade immune surveillance and contribute to metastatic potential, making them a key factor in assessing the efficacy of immunotherapies.

The advent of real-time imaging systems represents a pivotal advancement in biology and medicine, offering significant advantages over traditional endpoint assays such as chromium release assay, MTT assay, crystal violet staining, or flow cytometry, since it allows continuous monitoring of NK cell activity, revealing the dynamic processes of tumor cell death over extended periods. This also enables direct visualization of changes within 3D tumor models, providing a more comprehensive understanding of NK cell function [20,21]. In this protocol, we outline a robust methodology for quantifying the cytotoxic activity of NK cells against 3D tumor models of two distinct cancer types: cholangiocarcinoma and breast cancer using a live-cell imaging system. To assess NK cell-mediated cytotoxicity, cancer cells were labeled with carboxyfluorescein succinimidyl ester (CFSE) and co-cultured with NK cells at various effector-to-target (E:T) ratios. The progressive death of tumor cells is monitored by propidium iodide (PI) uptake, while key parameters such as spheroid size, area, and fluorescence intensity were continuously measured throughout the co-culturing period. Data were analyzed using Celleste (Thermo Fisher), GraphPad Prism, and ImageJ, a free, user-friendly, and widely accessible software for image analysis. This approach offers a precise and reliable platform for evaluating NK cell function in a physiological relevant 3D environment, providing valuable insights into their role in immune surveillance and potential therapeutic applications. Furthermore, this versatile approach can be readily adapted to other sources of NK cells such as those derived from induced pluripotent stem cells (iPSCs) or investigate the efficacy of other types of cancers or treatments, such as chemotherapy and combination therapies.

## Materials and methods

The protocol described in this article is published on protocols.io, DOI: https://dx.doi.org/10.17504/protocols.io.g2krbycv7, and is included for printing as supporting information file 1 with this article.

### Samples and ethics declarations

Human peripheral blood mononuclear cells (PBMCs) were isolated from whole blood leftover specimens obtained from leukocyte reduction system (LRS) cones provided by anonymous healthy donors. Blood collection followed the protocol approved by the Siriraj Institutional Review Board (SiRB; Protocol No. Si 493/2023) and complied with the Declaration of Helsinki (1975). Each donor received detailed study information, including a participant information sheet and an explanation of the research, and provided written informed consent. Recruitment for the study occurred between October 16 and November 3, 2023. Approximately 3 mL of blood was collected from each LRS cone for CD56^+^ cell isolation.

## Results

**Fig 1:**
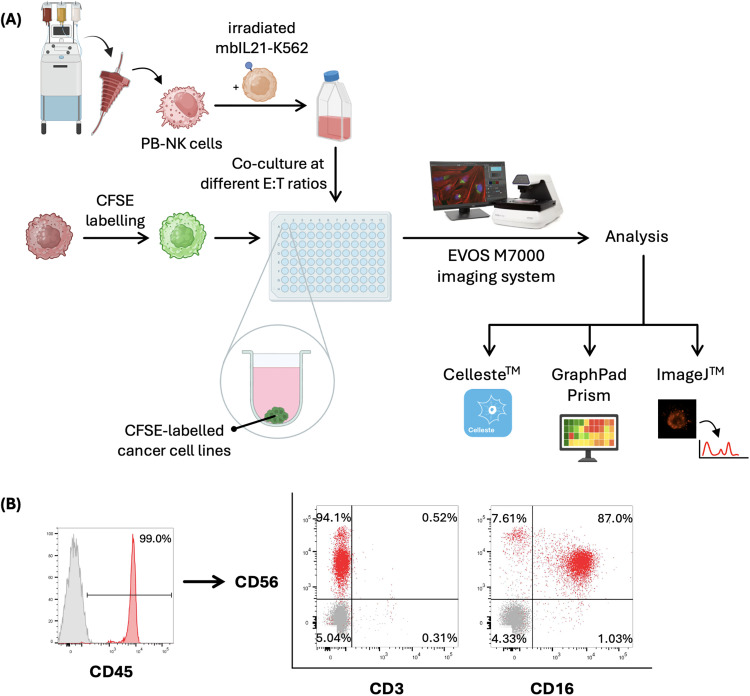
(A) Schematic diagram showing the experimental workflow of this study (B) Representative gating strategy for flow cytometric analysis of the expanded PB-NK cells.

In this protocol, the 3D spheroids of two solid cancer cell lines were used as a model to evaluate the dynamic cytotoxicity of NK cells (Fig 1A). PBMCs were isolated through density gradient centrifugation, followed by the sorting of CD56^+^ cells using magnetic separation. The isolated cells were then expanded using an irradiated, genetically modified membrane-bound (mb)-IL21 K562 myelogenous leukemic cell line in the presence of human IL-2. After 12 days of expansion, the phenotype of the expanded cells was analyzed using flow cytometry. The Boolean gating showed that approximately 95.1 ± 2.07% of the cells were positive for CD45 and CD56, and negative for CD3. Notably, 83.9 ± 4.25% of this population was positive for CD16, indicating the presence of the cytolytic NK cell population (Fig 1B) [4].

**Fig 2:**
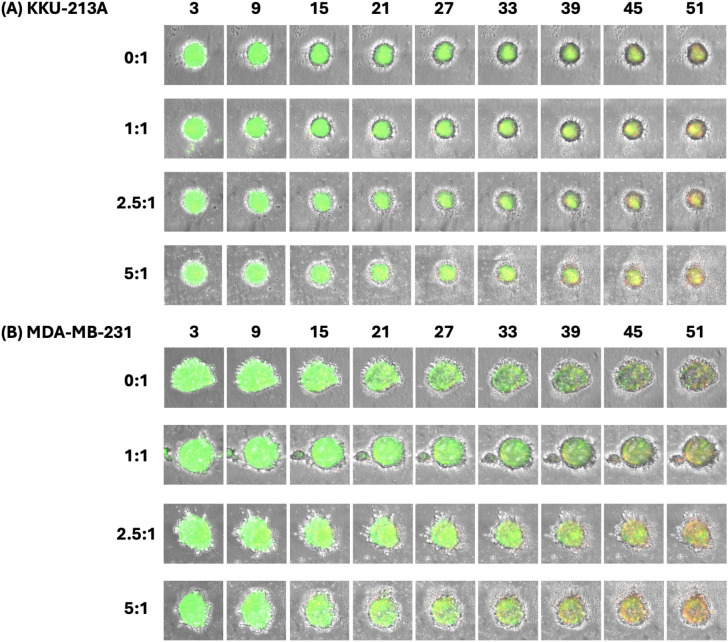
Morphology of the tumor spheroids (green) after being co-cultured with NK cells at different E:T ratios for 51 hours. Red color indicates the PI.

The dynamic cytotoxic activity of NK cells against tumor spheroids was evaluated using the EVOS M7000 imaging system. Two different cell lines representing aggressive solid cancer: KKU-213A (cholangiocarcinoma) and MDA-MB-231 (breast cancer), were used in the experiment. The tumor targets were stained with CFSE for tracking, while dead cells were stained with PI. The brightfield and fluorescence images were taken once every 6 hours (Fig 2). The progression of cell killing was observed by tracking the change in fluorescence intensity over time at different effector-to-target (E:T) ratios. From the brightfield images, we could notice the difference in morphology of the spheroids from different cancer cell lines. With the same spheroid formation method and seeding density, the spheroids of the KKU-213A cell line had spherical shapes with defined borders (Fig 2A), while the MDA-MB-231 spheroids were not perfectly round and had spike-like protrusion morphology (Fig 2B).

**Fig 3:**
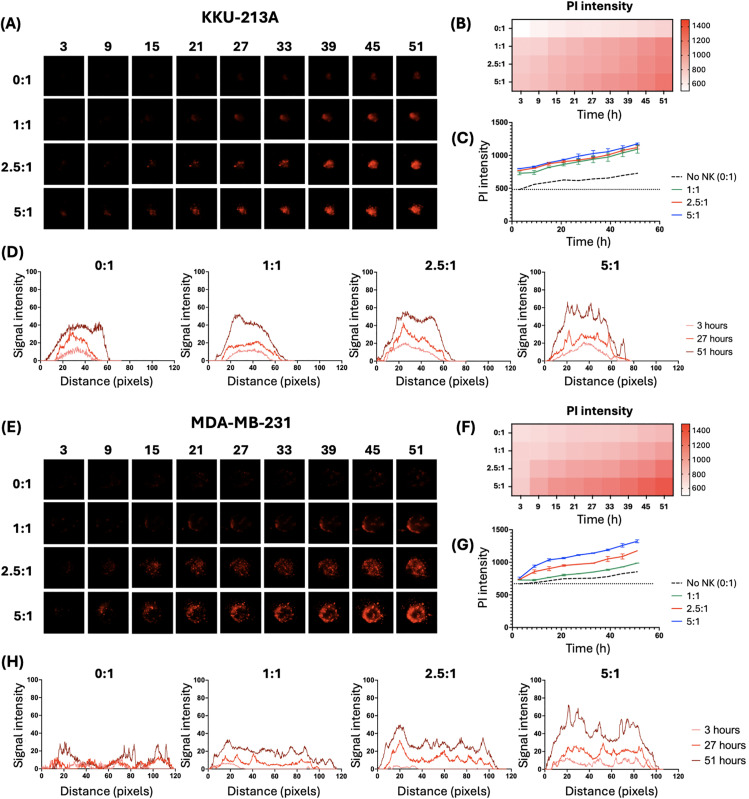
Analysis of cell death in KKU-213A (A – D) and MDA-MB-231 cell lines (E – H) after being co-cultured with NK cells over time. **(A and E)** Representative fluorescence images of KKU-213A and MDA-MB-231 spheroids stained with PI over time under various E:T ratios. **(B and F)** Heatmap summarizing the average PI fluorescence intensity across time points and E:T ratios (n = 3). **(C and G)** MFI of PI staining over time, showing dose-dependent cell death. Dashed lines: control (0:1, no NK cells); solid lines: E:T ratios of 1:1 (green), 2.5:1 (red), and 5:1 (blue). **(D and H)** The average PI intensity profiles across spheroids (n = 3).

We anticipated that the dissimilarity in spheroid morphology could lead to differences in dying patterns, with immune effector cells targeting them differently. Therefore, we focused on the changes in mean fluorescence intensity (MFI) of PI throughout the experiment. The data were measured by the intensity volume using the Celleste program. The dynamics of PI uptake served as a marker of cell membrane integrity loss, indicating cell death. As expected, both cell lines showed distinct cell death patterns. The KKU-213A spheroids started dying from the middle, while the MDA-MB-231 spheroids started from the edges (Figs 3A and 3E). Average PI intensity data of spheroids, illustrated by heatmaps (Figs 3B and 3F) and line graphs (Figs 3C and 3G), displayed similar trends in both types of spheroids, with lower fluorescence at earlier time points and lower E:T ratios, and higher fluorescence at later time points and higher ratios. The heatmap provides an intuitive overview of the trend across conditions, while the line graph offers more precise, quantitative insights with statistical variation. Together, they effectively demonstrate the relationship between E:T ratio, time, and cell death intensity. A closer examination of the PI signal intensity profiles revealed distinct behaviors between the two cell lines. As the plot illustrates the signal intensity of PI across the distance within a spheroid, it could provide a spatial profile of cell death over time. The KKU-213A spheroids exhibited narrower, more pronounced peaks concentrated at their centers, whereas the MDA-MB-231 spheroids exhibited broader profiles, with high peaks predominantly observed along their edges (Figs 3D and 3H). These differences underscore the variability in the susceptibility of the two cell lines to the treatment and may reflect differences in spheroid structure, cell death mechanisms, or treatment responses.

The data suggest that the NK cell cytotoxicity is time and dose-dependent, as evidenced by increasing PI fluorescent intensity. In addition, the differences in PI signal intensity profiles between the cell lines may reflect variations in their susceptibility to the treatments, offering insights into how different tumor types respond to therapeutic interventions. These findings could have important implications for understanding cell death mechanisms in diverse contexts and for optimizing treatment strategies tailored to specific cancer types.

**Fig 4:**
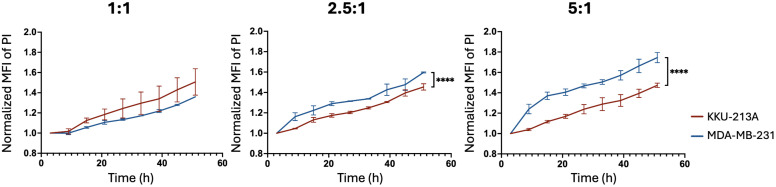
Comparison of normalized MFI of PI with significant differences indicated (**p < 0.01, ***p < 0.001, ****p < 0.0001). Error bars show mean ± SEM, n = 3, statistical analysis was performed by two-way ANOVA.

**Fig 5:**
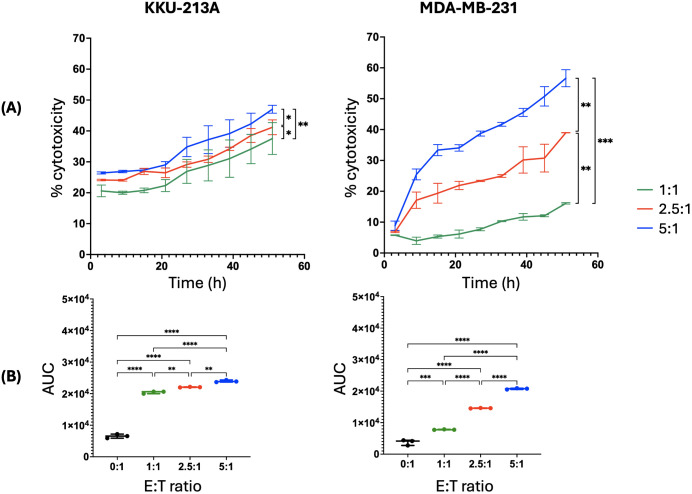
NK cell-mediated cytotoxicity against KKU-213A and MDA-MB-231 cell lines at varying E:T ratios. **(A)** Time-dependent percentage cytotoxicity. **(B)** AUC of MFI data with significant differences indicated (**p < 0.01, ***p < 0.001, ****p < 0.0001). Error bars show mean ± SEM, n = 3, statistical analysis was performed by two-way ANOVA.

While both cell lines exhibited time- and dose-dependent increases in PI fluorescent intensity, the KKU-213A spheroids demonstrated a significantly slower response to the treatment than the MDA-MB-231 spheroids, especially at higher E:T ratios (Fig 4). We found that the highest E:T ratio demonstrated the most substantial cytotoxic activity, with a rapid increase in specific killing, reaching approximately 47.0 ± 1.29% for KKU-213A and 56.6 ± 2.78% for MDA-MB-231 cells by 51 hours (Fig 5A). The specific killing percentage increased with higher E:T ratios. Additionally, we also calculated the area under the curve (AUC) to determine the overall killing efficacy of NK cells. The AUC data demonstrated significant differences in NK cell-killing activity in a dose-dependent manner (Fig 5B), consistent with the pattern observed in the calculation of percentage cytotoxicity.

To further strengthen these findings, we also extended the analysis to include other solid cancer cell lines; HeLa (cervical cancer) and A549 (lung adenocarcinoma). Spheroids could be formed from both cell lines, but with slight differences in morphology HeLa spheroids were larger and contained more defined border, while A549 spheroids were more compact (S1 Fig). The dynamics of NK cell cytotoxicity revealed differences between two spheroid models. In HeLa spheroids, PI uptake increased progressively in a dose- and time-dependent manner. The effectiveness of NK cells against HeLa spheroids was potent, showing robust killing activity as early as a few hours after co-culture, even at low E:T ratios. On the other hand, PI uptake in A549 spheroids was significantly lower than those of HeLa, but still progressed in a dose- and time-dependent manners. Robust effectiveness of NK cell cytotoxicity was found at high E:T ratios. Quantitative analyses also supported these observations. Heatmaps and line graphs demonstrated a clear difference in the dying patterns, further illustrating greater susceptibility of HeLa spheroids to NK cell-mediated killing. Consistent with this, the AUC analysis revealed significant increases in cytotoxicity with higher E:T ratios in both models (S2 Fig).

## Conclusion

This protocol highlights the real-time dynamics of NK cell-mediated cytotoxicity against tumor spheroids using live-cell imaging. Our data capture distinct morphological changes and dying patterns, clearly revealing that increasing the E:T ratios accelerates and amplifies tumor cell death. Notably, the KKU-213A cholangiocarcinoma cell line and HeLa cervical cancer cell line displayed higher sensitivity to NK cell activity compared to the MDA-MB-231 breast cancer cell line and A549 lung adenocarcinoma cell line. Despite differences in susceptibility to NK cell-mediated cytotoxicity, all cell lines demonstrated efficient killing at low E:T ratios and within a few hours of co-culture. We demonstrate multiple robust analysis approaches to assess immune effector cell cytotoxicity, including direct quantification of cytotoxicity percentages from PI intensity data and calculation of the AUC. Heatmaps and line plots effectively summarize spatial and temporal trends, while signal intensity variations reveal differences in cell death patterns between cancer cell lines. These visual and quantitative tools underscore the potential of live-cell imaging to evaluate NK cell efficacy in targeting diverse cancer types. Crucially, this protocol can be readily extended to evaluate dynamic NK cell-mediated cytotoxicity and dying patterns in other cancer types or treatments. To this end, this protocol provides a platform for studying cancer in a more physiologically relevant in vitro model, as tumor spheroids better mimic the 3D architecture, microenvironment, and cell-cell interactions of solid tumors compared to traditional 2D monolayer cultures. These models enhance our ability to investigate immune-cancer interactions and improve the predictive value of in vitro findings for therapeutic development.

## Supporting information

S1 File Step-by-step protocol, also available on protocol.io. (PDF)

S2 File Minimal data set including values behind mean, mean, and standard deviation. (XLSX)

S1 Fig Analysis of cell death in HeLa (A – D) and A549 cell lines (E – H) after being co-cultured with NK cells over time. (A and E) Representative fluorescence images of HeLa and A549 spheroids stained with PI over time under various E:T ratios. (B and F) Heatmap summarizing the average PI fluorescence intensity across time points and E:T ratios (n = 3). (C and G) MFI of PI staining over time, showing dose-dependent cell death. Dashed lines: control (0:1, no NK cells); solid lines: E:T ratios of 1:1 (green), 2.5:1 (red), and 5:1 (blue). (D and H) The average PI intensity profiles across spheroids (n = 3).
(TIFF)

S2 Fig AUC of MFI data with significant differences indicated (**p < 0.01, ***p < 0.001, ****p < 0.0001). Error bars show mean ± SEM, n = 3, statistical analysis was performed by two-way ANOVA.
(TIFF)
