# Supplementary material for: Quantification of timelapse 3D tumor spheroid killing activity of NK cells using a live-cell imaging system
Source: PLoS One. 2025 Oct 14;20(10):e0334246. doi: 10.1371/journal.pone.0334246 (PMC12520373; doi:10.1371/journal.pone.0334246)
Supplement: S1 File — (PDF) [file pone.0334246.s001.pdf]

Jun 05, 2025 Version 4

Quantification of timelapse 3D tumor spheroid killing activity of NK cells using a live-cell imaging system V.4

DOI
dx.doi.org/10.17504/protocols.io.g2krbycv7

Jakkrapatra Srisantitham[1,2], nontaphat.tho[1], Siriwal Suwanpitak[1], methichit.wat[1]

[1]Siriraj Center for Regenerative Medicine, Research Department, Faculty of Medicine Siriraj Hospital, Mahidol University, Bangkok, Thailand;
[2]Department of Immunology, Faculty of Medicine Siriraj Hospital, Mahidol University, Bangkok, Thailand

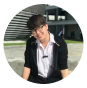

Jakkrapatra Srisantitham
Mahidol University

Create & collaborate more with a free account

Edit and publish protocols, collaborate in communities, share insights through comments, and track progress with run records.

Create free account

OPEN ACCESS

DOI: **https://dx.doi.org/10.17504/protocols.io.g2krbycv7**

Protocol Citation: Jakkrapatra Srisantitham, nontaphat.tho, Siriwal Suwanpitak, methichit.wat 2025. Quantification of timelapse 3D tumor spheroid killing activity of NK cells using a live-cell imaging system. **protocols.io**
https://dx.doi.org/10.17504/protocols.io.g2krbycv7 Version created by **Jakkrapatra Srisantitham**

License: This is an open access protocol distributed under the terms of the **Creative Commons Attribution License**, which permits unrestricted use, distribution, and reproduction in any medium, provided the original author and source are credited

Protocol status: Working
We use this protocol and it's working

Created: January 13, 2025

Last Modified: June 05, 2025

Protocol Integer ID: 219505

Keywords: NK cells, Live-cell imaging, Tumor spheroids, Immunotherapy, Cell therapy, assessment of nk cell, cytotoxic activities against 3d tumor spheroid, quantification of timelapse 3d tumor spheroid, 3d tumor spheroid, nk cell, activity of nk cell, timelapse 3d tumor spheroid, negative selection of peripheral blood nk, peripheral blood nk, cell imaging system the protocol, cell imaging, using flow cytometry, flow cytometry, cell imaging system, peripheral blood mononuclear cell, isolation of peripheral blood mononuclear cell, subsequent analysis of spheroid, imagej software, nk, cytotoxic activity, utilizing evos m7000 imaging system, spheroid, evos m7000 imaging system, cell

Funders Acknowledgements:
Faculty of Medicine Siriraj Hospital, Mahidol University, Thailand
Grant ID: R016837001
National Research Council of Thailand (NRCT)
Grant ID: N41A661130
National Research Council of Thailand (NRCT)
Grant ID: N42A660313

Abstract

The protocols for isolation of peripheral blood mononuclear cells (PBMCs), negative selection of peripheral blood NK (PB-NK) cells, expansion , and evaluation of PB-NK cells using flow cytometry are performed following our published work (1). Here, we describe the assessment of NK cell cytotoxic activities against 3D tumor spheroids, live-cell imaging utilizing EVOS M7000 imaging system, and the subsequent analysis of spheroid killing via Celleste, GraphPad Prism, and ImageJ software.

Materials

1.Culture of solid cancer cell lines

- Complete DMEM/F12 medium for cholangiocarcinoma cell line; DMEM/F12 (Gibco), 10% fetal bovine serum (Thermo Fisher), 2 mM GlutaMAX (Gibco), 1% penicillin/Streptomycin (Gibco)
- Complete DMEM medium for breast cancer cell line; DMEM (Gibco), 10% fetal bovine serum (Thermo Fisher), 2 mM GlutaMAX (Gibco), 1% penicillin/Streptomycin (Gibco)
- 0.1% Trypsin/EDTA (Gibco)
- Phosphate buffered saline (PBS)
- 0.4% Trypan Blue Solution (Gibco)
- Hemocytometer
- Transfer pipette
- 25-cm^2 cell culture flask
- Sterile pipette tips (200 and 1000 µL)
- Inverted microscope
- Tabletop centrifuge

2. Spheroid formation

- CFSE labeled-cholangiocarcinoma and breast cancer cell line
- Complete DMEM/F12 medium for cholangiocarcinoma cell line; DMEM/F12 (Gibco), 10% fetal bovine serum (Thermo Fisher), 2 mM GlutaMAX (Gibco), 1% penicillin/Streptomycin (Gibco)
- Complete DMEM medium for breast cancer cell line; DMEM (Gibco), 10% fetal bovine serum (Thermo Fisher), 2 mM GlutaMAX (Gibco), 1% penicillin/Streptomycin (Gibco)
- Matrigel matrix (Corning)
- Trypan Blue Solution, 0.4% (Gibco)
- Hemocytometer
- Ultra-low attachment round-bottom 96-well plate (Corning)
- 15-mL conical tube
- Transfer pipette
- Pipette tips (200 and 1000 µL)
- Inverted fluorescence microscope
- Tabletop centrifuge

3. Determination of 3D killing activity using a live-cell imaging system

- PB-NK cells
- PB-NK culture medium; RPMI-1640 (Gibco), 10% fetal bovine serum (Thermo Fisher), 2 mM GlutaMAX (Gibco), 1% non-essential amino acid (Gibco), 1% penicillin/Streptomycin (Gibco)
- CFSE-labelled cholangiocarcinoma and breast cancer cell lines
- Propidium iodide (PI, Thermo Fisher)
- 0.1% Triton X-100
- 15-mL conical tube
- Transfer pipette
- Pipette tips (200 and 1000 µL)
- EVOS M7000 imaging system
- Inverted fluorescence microscope

- Tabletop microscope

4. Analysis of live cell killing activity

- Celleste software (Thermo Fisher)
- GraphPad Prism software
- ImageJ software

Culture of solid cancer cell lines

1 Passaging solid cancer cell lines

Note

For the 3D tumor spheroid killing assay, we use KKU-213A and MDA-MB-231, representing aggressive cancer cell lines, as targets. KKU-213A cells are cultured in the complete DMEM/F12 medium, while MDA-MB-231 cells are cultured in the complete DMEM medium. Cells are passaged when they attain 70-80% confluence, typically occurring within 3-4 days, and are cultured in a 25-cm^2 culture flask.

1.1 Remove the culture medium and rinse the vessels with 1X PBS.

1.2 Add 1 mL of 0.1% trypsin/EDTA into the flask and incubate the cells at 37 °C, 5% CO_2 for 5 min.

1.3 After the incubation, add 4 mL of complete medium and thoroughly resuspend the cells using a serological pipette.

1.4 Transfer the cell suspension to a 15-mL conical tube.

1.5 Centrifuge the cells at 500 × g at room temperature for 5 min.

1.6 Discard supernatant and resuspend the cells with 1 mL of complete medium.

1.7 Perform cell counting using the trypan blue exclusion assay.

1.8 Transfer 2×10^5 viable cells into a new 25-cm^2 culture flask containing 5 mL of complete medium and incubate the cells at 37 °C, 5% CO_2.

Spheroid formation

2 CFSE labeling

2.1 Perform cell dissociation as described in steps 1.1 to 1.7.

2.2 Transfer 2 × 10^5 viable cells to a 15-mL conical tube, wash with 1X PBS.

2.3 Centrifuge the cells at 500 × g at room temperature for 5 min.

2.4 Discard the supernatant and resuspend the cells with 1 mL of 1X PBS.

2.5 Add CFSE solution to the final concentration of 2.5 µM, vortex briefly and incubate at 37 °C, 5% CO_2 for 20 min in the dark.

2.6 After incubation, add 5 mL of complete cancer media to the cell suspension.

2.7 Centrifuge the cells at 500 × g at room temperature for 5 min.

2.8 Discard the supernatant and resuspend the cells with 1 mL of culture media.

3 Formation of tumour spheroids

3.1 Prepare cancer culture media containing 2.5% Matrigel matrix and chill the media on ice.

3.2 Aliquot appropriate cell number of the CFSE-labelled cholangiocarcinoma or breast cancer cells to a 15-mL conical tube.

3.3 Centrifuge the cells at 500 × g at room temperature for 5 min.

3.4 Discard the supernatant.

3.5 Resuspend the CFSE-labelled cholangiocarcinoma or breast cancer cells with pre-chilled culture media containing 2.5% Matrigel matrix.

3.6 Transfer the CFSE-labelled cholangiocarcinoma or breast cancer cells onto an ultra-low attachment round-bottom 96-well plate at a seeding density of 2×10^3 cells/100 µL/well.

3.7 Centrifuge the plate at 1,000 × g for 10 min at 4 °C.

3.8 Incubate the spheroids at 37 °C, 5% CO_2 for 2 days.

Determination of 3D killing activity using a live-cell imaging system

4 Co-culture of PB-NK cells with tumour spheroids

4.1 After 2 days of spheroid formation, thaw PB-NK cells in the PB-NK cell medium.

4.2 Transfer the thawed PB-NK cells to a 15-mL tube and centrifuge at 500 × g at room temperature for 5 min.

4.3 After centrifugation, remove the supernatant, resuspend the cell pellet in 1 mL of PB-NK culture medium, and portion the necessary number of PB-NK cells for all co-culture condition into a 15-mL conical tube.

4.4 Centrifuge the cells at 500 × g at room temperature for 5 min.

4.5 Discard the supernatant and resuspend the cell pellet with an appropriate volume of PB-NK culture medium without IL-2 supplementation.

4.6 Add propidium iodide (PI) to the concentration of 2 µg/mL and mix thoroughly using an autopipette

Note

We recommend performing at least 3 replicates for each E:T ratio, therefore, prepare approximately 5 mL of PB-NK medium containing 2 µg/mL PI.

.

4.7 Add 100 µL of the suspended PB-NK cells to a well of the ultra-low attachment round-bottom 96-well plate containing tumour spheroids (from step 3.8) with cell numbers adjusted according to various effector-to-target (E:T) ratios; for this protocol, E:T ratios of 0:1, 1:1, 2.5:1, and 5:1, are utilised, therefore, the NK cell number should be 2×10^3, 5×10^3, and 1×10^4 cells/well for 1:1, 2.5:1, and 5:1 ratio, respectively.

Note

Carefully add the PB-NK cells to prevent disruption of the spheroids. The total volume of culture medium per well is 200 µL, with the final concentration of PI being 1 µg/mL.

4.8 Centrifuge the 96-well plate at 1,000 × g for 10 min at 4 °C.

4.9 Add 0.1% Triton X-100 to the positive control wells to assess the maximum level of death.

Note

The maximum PI intensity of the positive control could be observed within the first 6 hours of co-culture.

4.10 Incubate the cells at 37 °C, 5% CO_2 in the EVOS M7000 imaging system and record the data for three days.

Note

Prepare control wells containing only the tumour spheroids (0:1 ratio) to determine the baseline level of spontaneous tumour cell death.

Analysis of live cell killing activity

5 Mean fluorescence intensity data acquisition using Celleste software

5.1 Collect data from 51 hours of co-culture from the EVOS M7000 imaging system.

5.2 Open the files in the Celleste program v5 by clicking ‘Open Images as Sequences’.

5.3 Select all files that include “..._z0...” and select ‘Group Related Files’ and ‘Open As Sequences’, then click ‘Open’

Note

This process might take some time, depending on the power of the hardware.

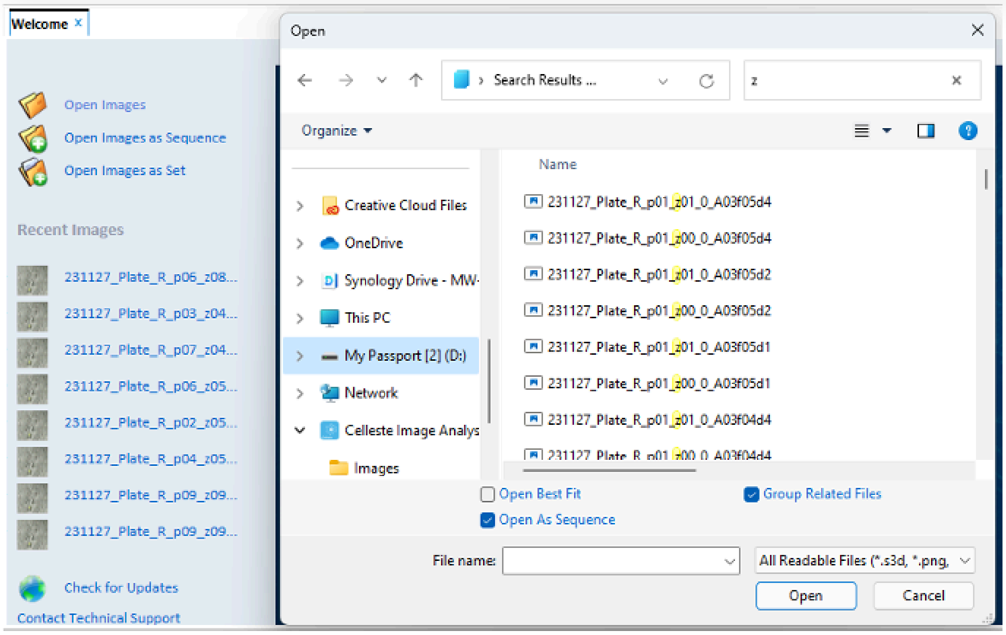

Figure 1 File selection for analysis

5.4 Click ‘Add Volume Measurement’ and select the channel for analysis, in this case, “RFP_Tiled” is used for analyzing PI intensity. The green area will appear in the interested area.

Note

This program allows analysis of several channels, including brightfield. In this protocol, we use PI and CFSE, thus we analysed only RFP and GFP channels.

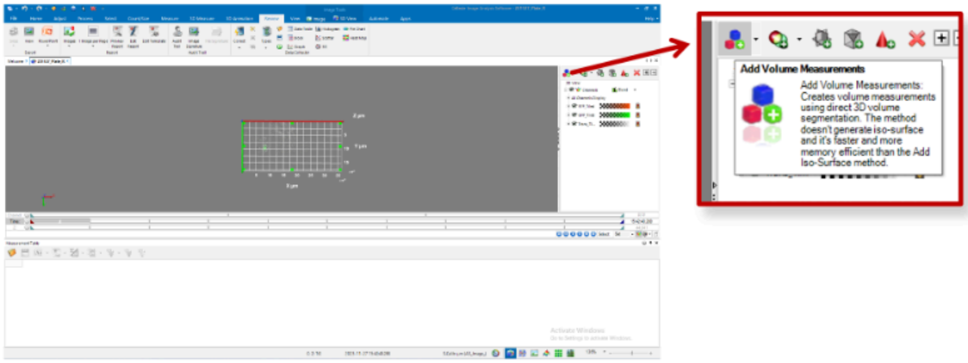

Figure 2 ‘Add Volume Measurements’ button for creating 3D volume measurements.

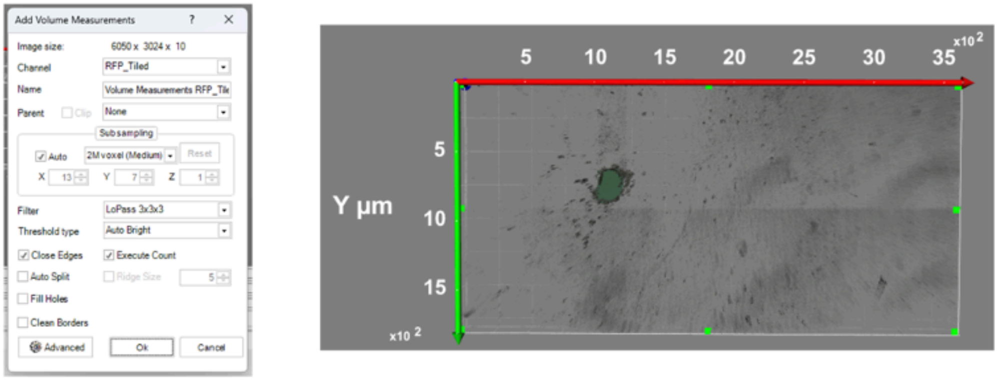

Figure 3 Pop-up window after creating volume measurement.

5.5 Adjust the RFP from the last time point to maximise the area of PI intensity.

5.6 Adjust GFP from the first time point to maximise the area of CFSE intensity by selecting “GFP_Tiled” (same menu as in step 5.4).

Note

The volume of RFP and GFP should not be much different.

5.7 Click on ‘3D Measurement’, then click ‘Edit Measurement Range’

5.8 Adjust “Volumes: Volume” and “Volumes: Density (mean)” to correct the size of only spheroids, not other trace objects in the wells.

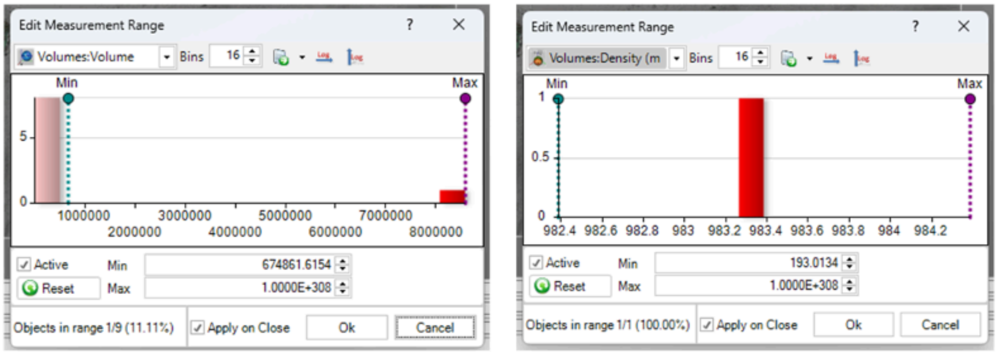

Figure 4 'Edit Measurement Range' window for adjusting the volume of spheroids to be analyzed.

5.9 Adjust the range of both GFP and RFP to be the same within the same well.

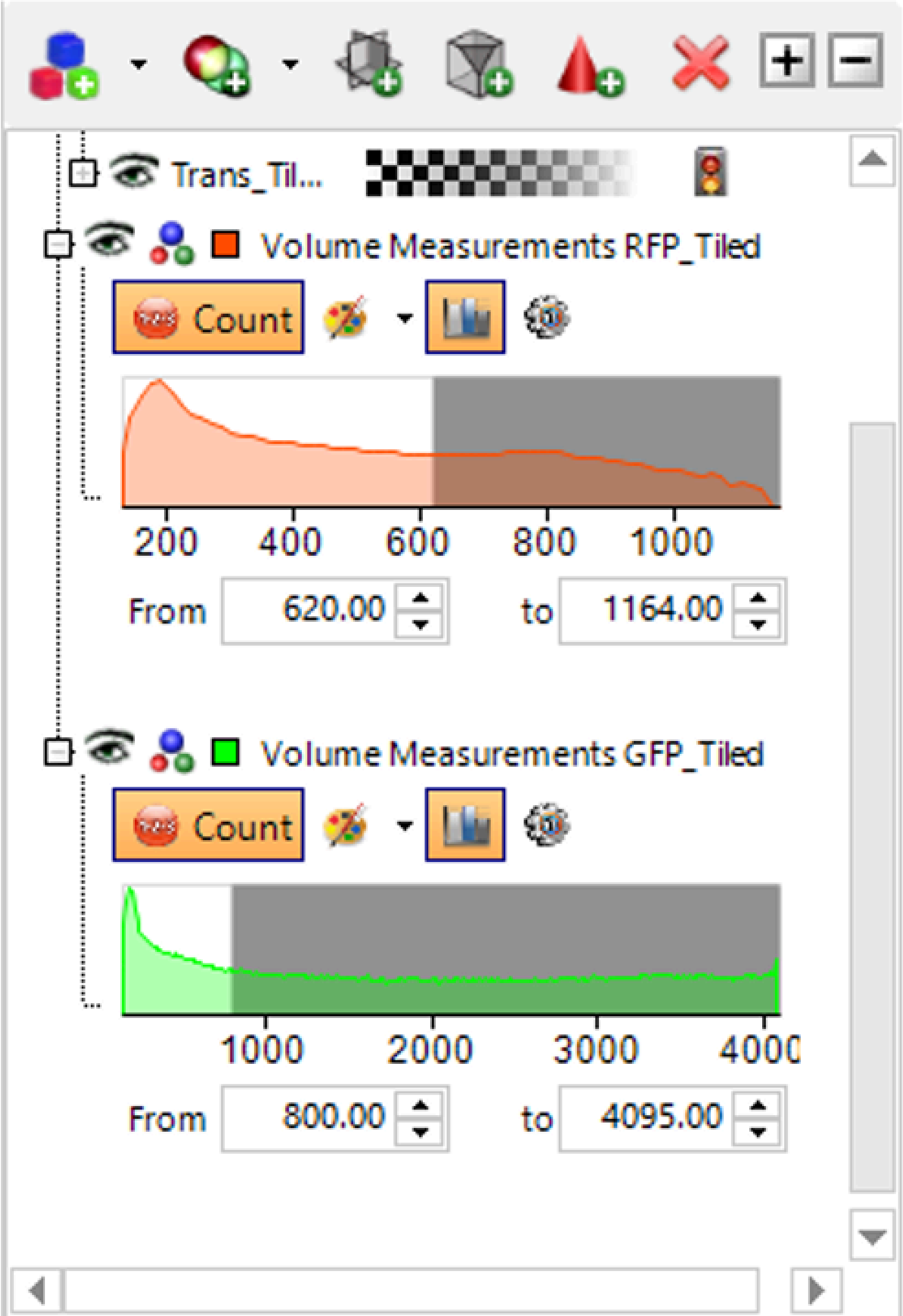

Figure 5 Adjustment of red and green are for analysis.

5.10 Click 'Show Data Table', then export the data to Microsoft Excel.

6 Strategy 1: Visualisation of dying position within the tumor spheroids upon co-culturing with NK cells using ImageJ software

Note

The dying position within the spheroids can be visualized to present different dying patterns of cancer cells. We use ImageJ software to analyze the fluorescent images taken from EVOS M7000. The brightfield images from 3 timepoints (first, middle, and final timepoints) are used to gate regions of interest (ROI) for analyzing the intensity profiles of PI intensity in each spheroid.

6.1 Open the brightfield image of spheroid at the final timepoint (51 hours) in the ImageJ software.

6.2 Use the ‘straight line’ tool to choose only the area of interest within a spheroid.

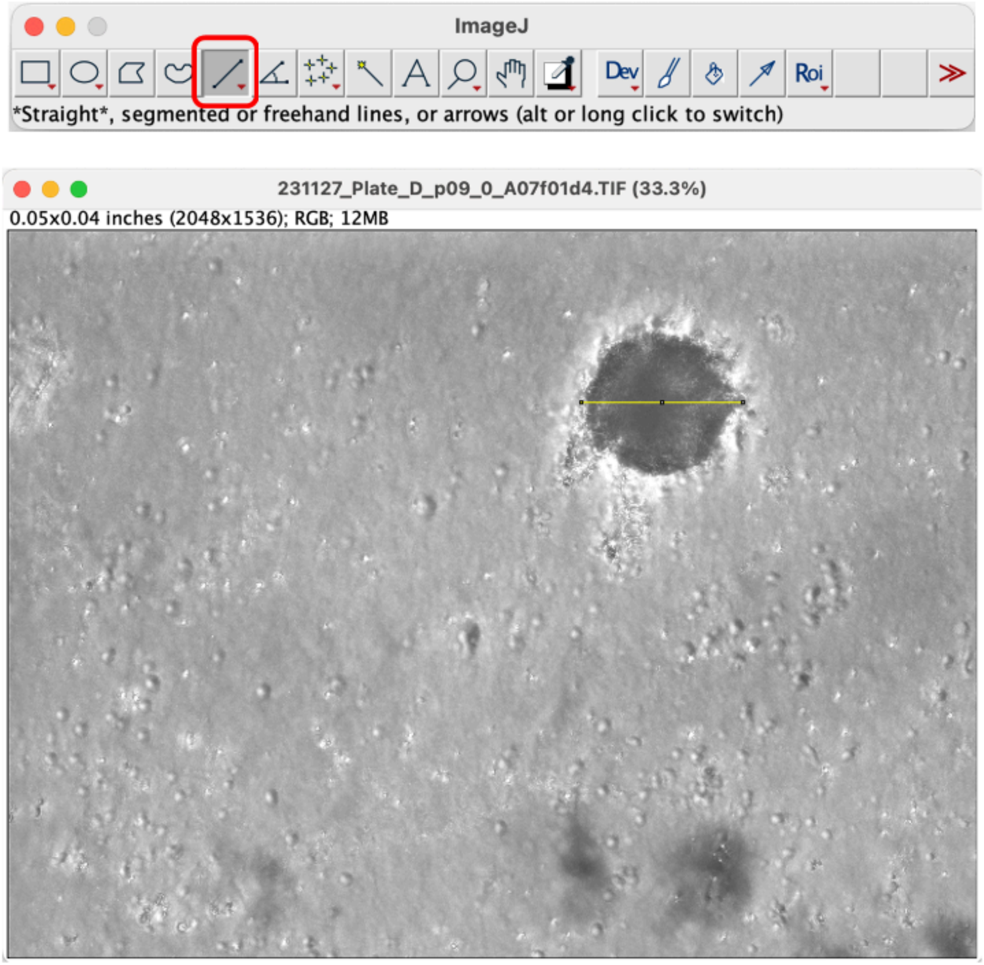

Figure 6 Selecting 'straight line' tool in ImageJ software and gating of spheroid area using brightfield image.

6.3 Select ‘Analyse’, then choose the ROI manager to record the ROI.

6.4 Rename the ROI to be the name of the selected well and timepoint.

Note

Saving the ROI is recommended by clicking 'More", then 'Save'.

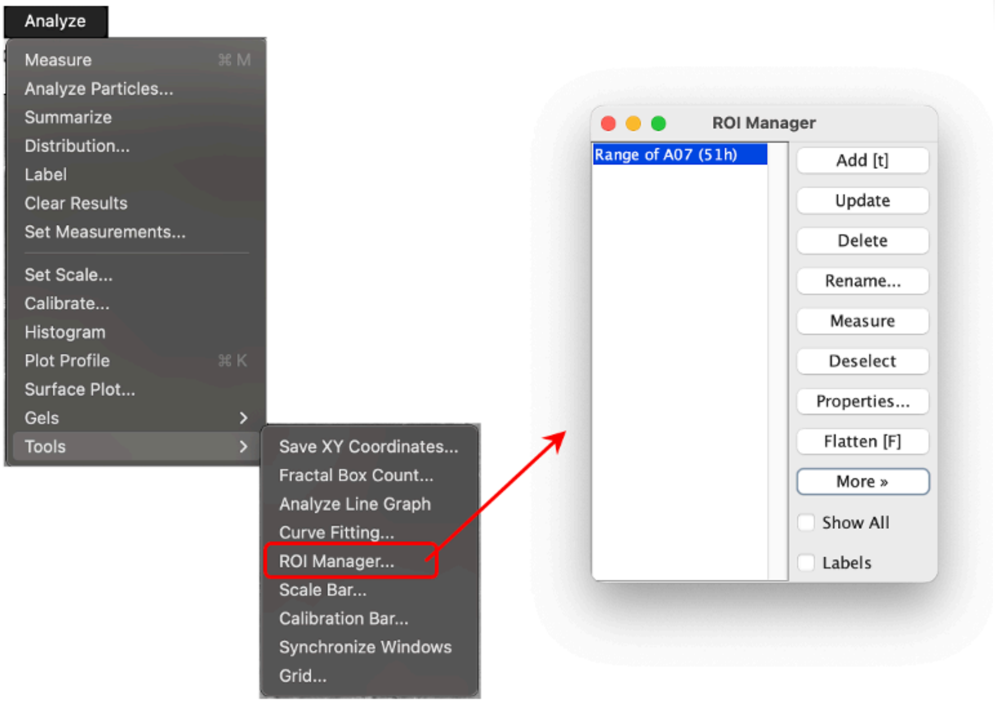

Figure 7 ROI manager menu for selecting and recording range of interest.

6.5 Open the spheroid image of the final timepoint (51 hours) with only red fluorescence protein (RFP) channel.

6.6 Click on the selected ROI from the same timepoint and click 'Add' to apply ROI to the spheroid image.

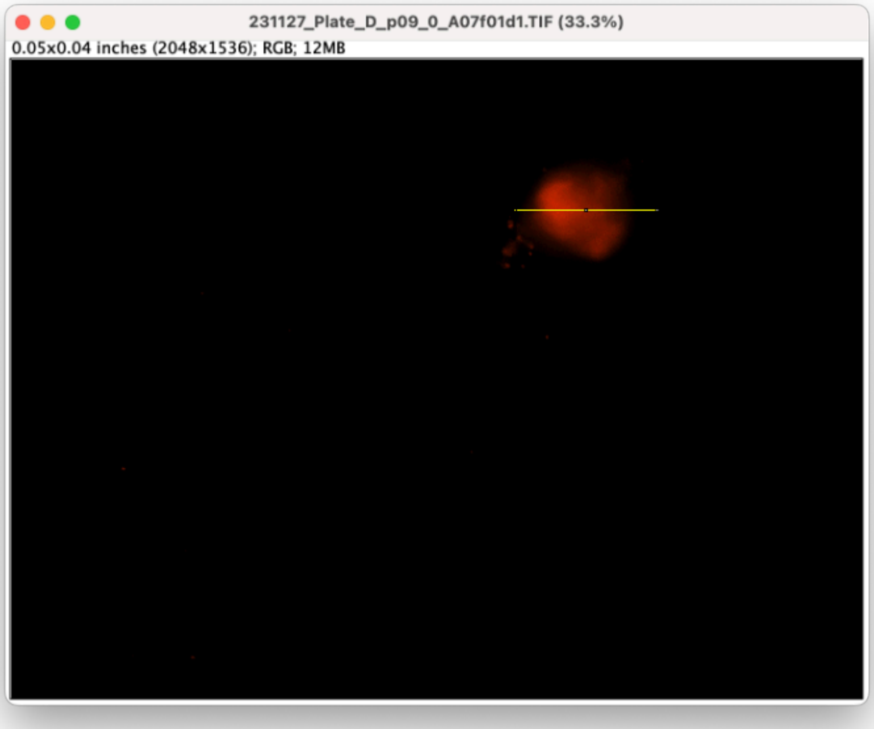

Figure 8 Applying the gate for ROI manager into the image with red fluorescence of PI.

6.7 Select ‘Analyse’, then choose ‘Plot Profile’ to generate the plot between signal intensity and distance of the ROI.

Note

The axis title may differ from the figure, the axis can be adjusted by clicking ‘More’, then ‘Axis Options’.

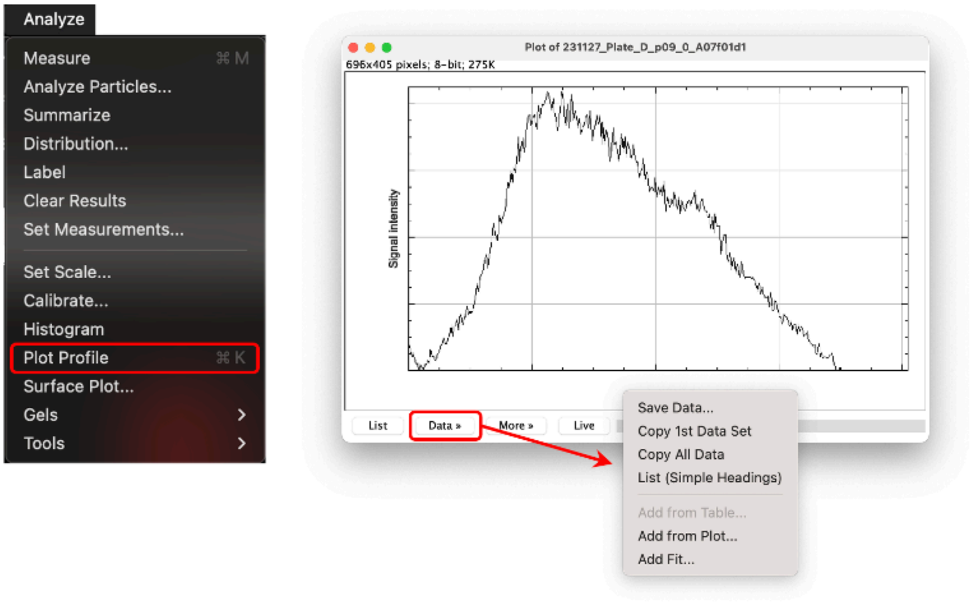

Figure 9 Analysis of the fluorescent profile using 'Plot Profile' menu.

6.8 To export the data of intensity profile, select ‘Data’, then ‘Copy All Data’.

6.9 Paste the data in GraphPad Prism to generate the plot from different timepoints.

Note

Different timepoints require at least three independent spheroids.

6.10 Perform the analysis in all timepoints from three different spheroids and plot them together in GraphPad Prism.

7 Strategy 2: Determination of cytotoxic activity using MFI of PI

7.1 From the MFI data ontained from step 4.1, percentage of specific killing of the PB-NK cells can be quantified using the following formula:

$$\% \text{ specific killing} = \frac{(\text{Experimental MFI} - \text{Spontaneous MFI})}{(\text{Maximum MFI} - \text{Spontaneous MFI})} \times 100$$

Note

The experimental MFI represents the mean fluorescence intensity of PI in the presence of effectors at a given E:T ratio, whereas the spontaneous MFI denotes the MFI of PI in wells containing only tumor spheroids to determine baseline spontaneous tumor cell death. Maximum MFI indicates the MFI of PI in wells with tumor spheroids treated with 0.1% Triton X-100 to ascertain the maximum level of cell death. This calculation provides a quantitative measure of the cytotoxic potential of PB-NK cells against the tumor spheroids.

7.2 Export the killing percentage data of every timepoints of three independent spheroids from all E:T ratios to GraphPad Prism.

7.3 Generate the plot between time (hour) and killing percentage to see NK cytotoxicity over time.

8 <u>Strategy 3: Determination of overall cytotoxicity using area under the curve</u>

Note

In this strategy, we use GraphPad Prism software to analyze the PI intensity data obtained from the previous part . Using this analysis, we can determine the overall killing activity of NK cells against different E:T ratios and different cancer types.

8.1 Import the data of PI intensity from the previous part to GraphPad Prism software.

Note

We recommend using the XY table format.

8.2 Generate a line graph showing the change in PI intensity over time.

8.3 Analyse the data by generating area under the curve (AUC).

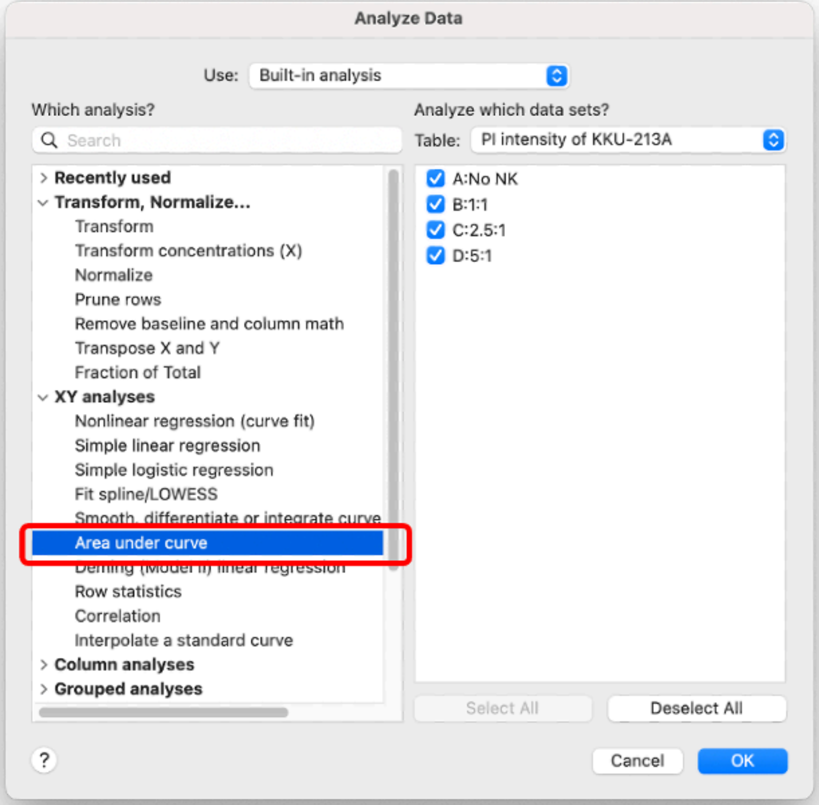

Figure 10 Analysis of area under the curve using Analysis menu in GraphPad Prism software.

8.4 Copy the total area displayed in the analysis panel and paste them in a new data table. The column graph should be seen in the graph area.

Protocol references

1. Thongsin N, Wattanapanitch M. A three-dimensional immune-oncology model for studying in vitro primary human NK cell cytotoxic activity. PLoS One. 2022;17(3):e0264366

Acknowledgements

The authors would like to thank Gibthai Co.Ltd for providing the EVOS M7000 imaging system and technical support.J.S. is supported by Siriraj Graduate Scholarship, Faculty of Medicine Siriraj Hospital, Mahidol University. M.W. is supported by Chalermphrakiat Grant, Faculty of Medicine Siriraj Hospital, Mahidol University.
