# Supplementary figures and images for: Quantification of timelapse 3D tumor spheroid killing activity of NK cells using a live-cell imaging system
Source: PLoS One. 2025 Oct 14;20(10):e0334246. doi: 10.1371/journal.pone.0334246 (PMC12520373; doi:10.1371/journal.pone.0334246)

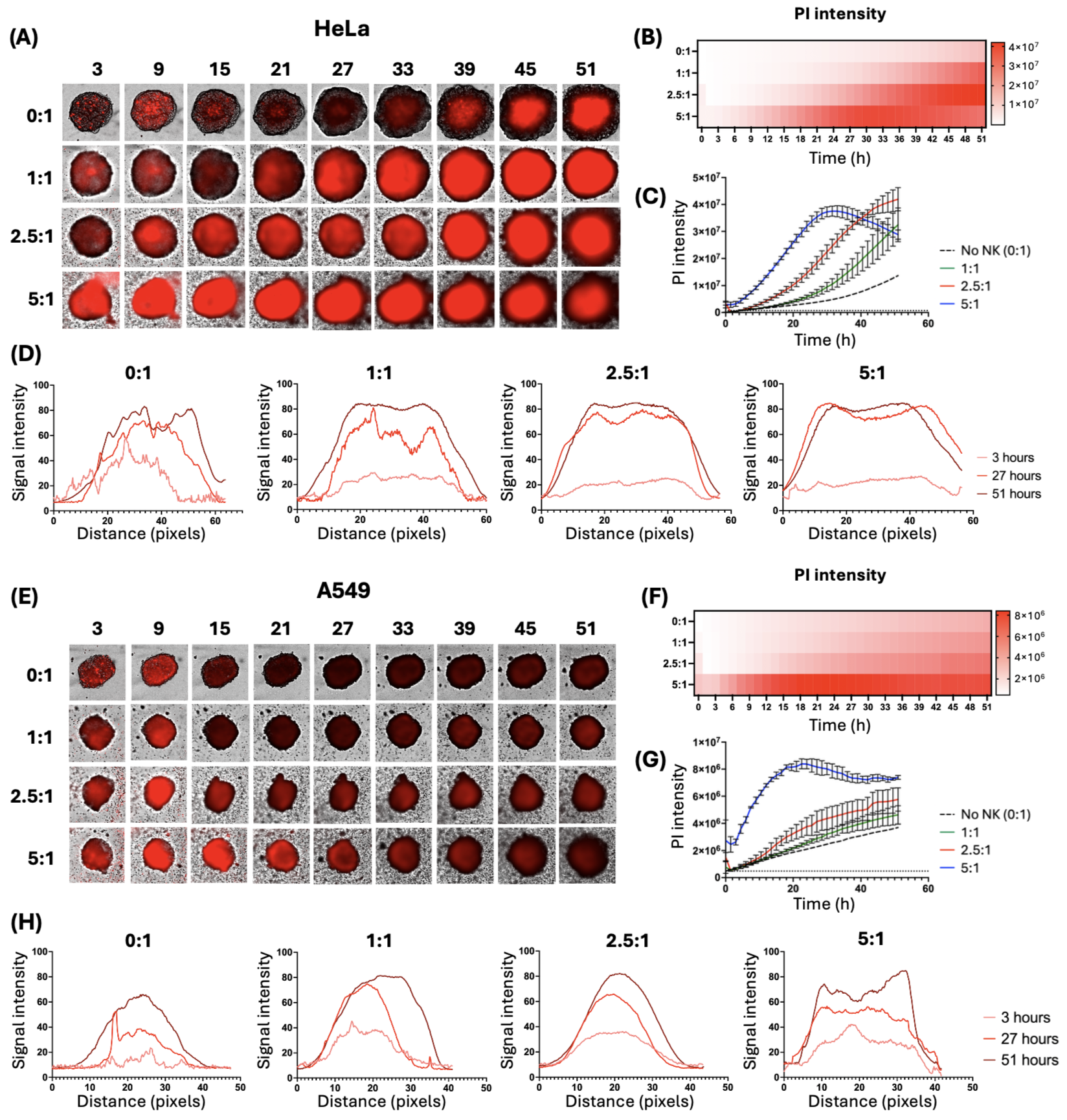

Supplement: S1 Fig — (A and E) Representative fluorescence images of HeLa and A549 spheroids stained with PI over time under various E:T ratios. (B and F) Heatmap summarizing the average PI fluorescence intensity across time points and E:T ratios (n = 3). (C and G) MFI of PI staining over time, showing dose-dependent cell death. Dashed lines: control (0:1, no NK cells); solid lines: E:T ratios of 1:1 (green), 2.5:1 (red), and 5:1 (blue). (D and H) The average PI intensity profiles across spheroids (n = 3). (TIFF) [file pone.0334246.s003.tiff]

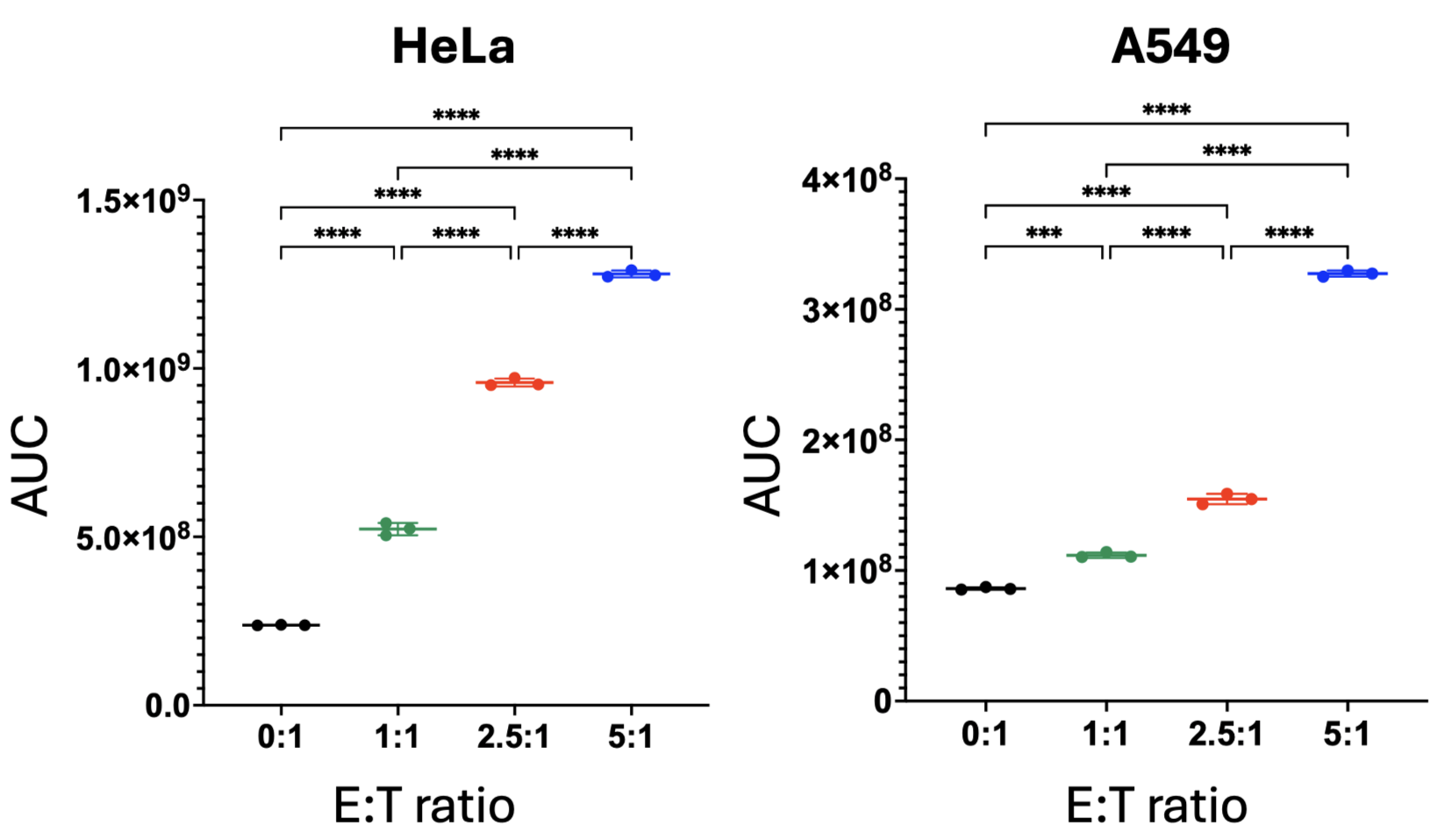

Supplement: S2 Fig — Error bars show mean ± SEM, n = 3, statistical analysis was performed by two-way ANOVA. (TIFF) [file pone.0334246.s004.tiff]
